# Microsecond-pulsed nanocalorimetry: a scalable approach for ultrasensitive heat capacity measurements

**DOI:** 10.1038/s41378-026-01426-7

**Published:** 2026-09-20

**Authors:** Hugo Gómez-Torres, Manel Molina-Ruiz, Simone Privitera, Enric Menéndez, Llibertat Abad, Jordi Sort, Olivier Bourgeois, Javier Rodriguez-Viejo, Aitor Lopeandia

**Affiliations:** 1 Catalan Institute of Nanoscience and Nanotechnology (ICN2), CSIC and BIST, Bellaterra, E-08193 Spain; 2 Departament de Física, Universitat Autònoma de Barcelona, E-08193 Bellaterra, Spain; 3 Institut de Microelectrònica de Barcelona (IMB-CNM-CSIC). Campus de la UAB, E-08193 Cerdanyola del Vallès (Barcelona), Spain; 4 Institució Catalana de Recerca i Estudis Avançats (ICREA), Pg. Lluís Companys 23, Barcelona, E-08010 Spain; 5 Université Grenoble Alpes, Institut Néel - CNRS UPR2940, 25 rue des Martyrs, BP 166, 38042, Grenoble, Cedex 9 France; 6 Present Address: Department of Physics, University of California, Berkeley, 94720 CA USA

**Keywords:** Electronic properties and materials, Nanometrology, Sensors

## Abstract

We introduce a nanocalorimetric technique based on microsecond-pulsed heating (µs-PHnC) that enables high-sensitivity, quasi-isothermal heat capacity measurements on nanoscale samples. Such resolution is critical for exploring thermodynamic signatures in low-dimensional materials, where conventional techniques fall short. By confining thermal excitation to microsecond timescales, this approach minimizes lateral heat diffusion, reduces heat capacity addenda to below 10⁻⁹ J K^-1^, and achieves noise densities as low as 75 pJ K⁻¹ √Hz mm⁻², unlocking precise thermodynamic characterization of subnanogram samples in areas as small as 30 × 30 µm². The method delivers exceptional temperature homogeneity, as demonstrated by resolving sharp phase transitions, such as the antiferromagnetic transition in ultrathin CoO films, with unprecedented clarity. Its quasi-static operation is inherently compatible with external stimuli, including magnetic and electric fields, thereby expanding its utility for in-operando thermodynamic studies. The method is scalable in lateral dimension because the microsecond excitation window confines the thermal diffusion length, allowing the effective calorimetric volume to shrink with the device footprint. This advancement establishes a robust and scalable platform for probing thermal phenomena in nanostructured and low-dimensional materials, significantly broadening the scope of nanocalorimetry.

## Introduction

The scaling down and production of materials with nanometric dimensions has revealed profound size-dependent effects in their thermodynamic behavior, including shifts in phase transition temperatures, altered enthalpic landscapes, and modified heat capacities^1,2^. These phenomena are particularly relevant in ultrathin films, nanostructures, and low-dimensional systems, where surface-to-volume ratios are high and energy exchanges are minimal. Accessing such thermodynamic quantities (heat capacity, enthalpy, and entropy changes) requires calorimetric techniques capable of resolving minute thermal signals. However, conventional calorimetry, which relies on bulk sample masses to ensure sufficient signal-to-noise ratio (SNR), is inherently limited in this context. The typical sample requirements and thermal inertia of traditional setups render them unsuitable for nanomaterials, which are often available only in nanogram quantities and exhibit low-energy transitions that are easily masked by background noise^3,4^.

Chip calorimetry has emerged as a powerful technique to overcome the limitations of conventional calorimetry, enabling the measurement of thermal properties in ultrathin films and low-dimensional systems with sample masses in the nanogram range or even below^4–6^. The miniaturization of calorimetric cells (CCs), enabled by advances in microfabrication, has led to the development of membrane-based devices characterized by ultralow heat capacity addenda and excellent thermal coupling between the sample, heater, and sensors. As first noted in early studies of low-temperature calorimetry, the background signal (proportional to the total heat capacity of the system) can obscure the thermal signature of small samples if the addenda is not minimized^7^. Reducing the heat capacity of the calorimetric platform and matching it to that of the sample significantly enhances the effective SNR. Furthermore, the improved thermal contact in membrane-based chips nearly eliminates thermal lag and allows for much faster heating and cooling rates. Since the calorimetric signal, defined under adiabatic conditions as the measurable temperature rise ΔT_CC_(t) in response to a known input power, scales with the heating rate, increasing the scan rate directly improves sensitivity. However, the scan rate must remain compatible with the intrinsic thermal diffusion timescales of both the sample and the calorimetric cell to ensure accurate measurements.

While existing nanocalorimetric techniques can characterize nanostructures with high sensitivity, they typically lack spatial (x-y) resolution and offer only depth information. In contrast, the proposed µs-PHnC technique overcomes this limitation by enabling local and selective characterization in the lateral (x-y) plane, allowing for precise thermal analysis of microscale features.

Three main nanocalorimetric methods have been implemented for chip calorimetry: AC calorimetry^7–10^, relaxation calorimetry^2,6,11^, and fast scanning calorimetry, which includes both custom microfabricated devices^4,12^ and commercial implementations such as the Flash-DSC developed by Schick^13^ and Mettler-Toledo. Flash-DSC, for instance, achieves high scan rates and can measure nanogram-scale samples, but its resolution is insufficient to capture subtle thermal events in ultrathin films and it remains limited by thermal diffusion length and platform design. In this work, we will focus on custom microfabricated devices used with fast scanning calorimetry, a technique often referred to as quasi-adiabatic nanocalorimetry (QAnC), since the microsecond-pulsed-heating nanocalorimetry technique presented in this paper is developed from QAnC principles.

AC-calorimetry: Building on the original concept introduced by Corbino in 1910, who proposed determining the specific heat of solids at high temperatures using alternating currents^8^. Sullivan and Seidel later established the theoretical foundation for steady-state AC calorimetry^10^. This approach has since been adapted to nanoscale systems and implemented in various nanocalorimeters, enabling high-sensitivity measurements of the heat capacity of mesoscopic and low-dimensional materials. As a steady-state method, AC calorimetry permits the determination of heat capacity under quasi-isothermal conditions, either by combining it with temperature ramps or by probing its dependence on external variables such as magnetic field, electric field, time, or pressure. Notable examples include the attojoule-resolution calorimetry of mesoscopic superconducting loops^9^. Although the oscillatory nature of AC calorimetry enables the use of lock-in detection strategies that significantly enhance sensitivity, a key limitation of the technique is the requirement to operate below the characteristic thermal diffusion frequency of the device and its surroundings. This constraint ensures thermal equilibrium between the heater and sensor but inherently limits the maximum achievable heating rate and, consequently, the amplitude of the calorimetric signal. Strategies involving single-element heater-sensor configurations may improve internal thermalization times; however, they require the detection of higher-order harmonics, which are strongly attenuated and highly susceptible to noise. While this trade-off reduces the overall sensitivity, it enables the lateral sensing area to be minimized to the micrometer scale, making the technique suitable for spatially resolved studies in highly confined systems ^14^.

Relaxation calorimetry: A time-domain technique in which a sample is heated by a small, known amount, and the subsequent temperature relaxation back to equilibrium is monitored. The heat capacity is determined from the time constant of the exponential decay together with the thermal conductance of the system. If the thermal conductance between the cell and its surroundings is well-characterized, the heat capacity of the cell can be accurately determined. In this approach, the calorimetric signal remains relatively weak and, similar to the maximum achievable heating rates, is limited by the system’s characteristic relaxation time. Nevertheless, the simplicity of this technique renders it highly scalable. Since its early implementation in 1972 by Bachmann et al^2^. through its adaptation in the first chip-based calorimeter^6^ to its recent application in the measurement of 2D materials such as graphene with heat capacity resolutions down to 10^−19 ^J/K^11^, relaxation calorimetry has proven to be a versatile and powerful tool for thermal characterization at the micro- and nanoscale. However, although long thermal relaxation times are generally not ideal for fast or highly localized measurements, they become essential at very low temperatures. In this regime, the thermalization time between electrons and the phonon bath increases significantly, as discussed by Roukes^3^. For this reason, relaxation calorimetry remains the preferred technique for heat capacity measurements under such conditions at very low T. A prominent example is its implementation in the Physical Property Measurement System (PPMS) developed by Quantum Design, which enables precise calorimetric measurements across a wide temperature range.

Quasi-adiabatic nanocalorimetry (QAnC): Among the techniques implemented in chip calorimetry, QAnC stands out for offering the highest sensitivity per unit analysis area, with values down to 15 pJ K⁻¹ mm⁻². Originally developed by Leslie Allen and co-workers^4^, it has since been implemented by many other research groups^4,12,13,15–18^. In this method, the calorimetric cell is rapidly heated several hundred degrees by Joule effect in few milliseconds using a single metallic element that acts as both heater and thermometer. The planar geometry and low thermal mass of the calorimetric cell enable high heating rates up to 10⁵ K/s, while maintaining moderate thermal homogeneity across the sensing area. Under high vacuum conditions, the calorimetric cell behaves adiabatically during the heating pulse, and the temperature evolution can be directly related to the input power. This configuration allows for the detection of subtle thermal events in ultrathin films with exceptional resolution ^19–21^.

However, the spatial resolution of QAnC is ultimately limited by the lateral thermal diffusion length, which defines the minimum effective analysis area sensed and, consequently, the minimal heat capacity addenda. When the analysis area approaches or falls below this limit, lateral heat spreading reduces temperature homogeneity and compromises the accuracy of the measurement. This limitation becomes particularly critical when attempting to measure individual samples that can only be fabricated or isolated with sufficient quality over restricted surface areas, as is often the case with certain two-dimensional (2D) materials.

Scaling down existing nanocalorimetric techniques often requires a trade-off between thermal homogeneity and measurement sensitivity. To address this, we present microsecond-pulse heating nanocalorimetry (µs-PHnC), a variant of quasi-adiabatic nanocalorimetry (QAnC) adapted to the microsecond timescale. The short duration of the pulses ensures that the temperature rise remains confined to the sensing region, preserving the quasi-adiabatic conditions required for direct heat capacity determination. This method retains the high sensitivity and temporal resolution of QAnC while incorporating several advantages of AC and relaxation calorimetry, namely, the ability to operate under quasi-isothermal conditions and to average over multiple pulses to reduce noise. Additionally, it enables the downscaling of the calorimetric cell to micrometer dimensions, but also shares some of the limitations of AC and relaxation techniques, such as reduced accuracy in measuring first-order transitions and limited capability for kinetic studies.

## The μS-pulsed nanocalorimetry

### Methodology and device design

**Fig. 1:**
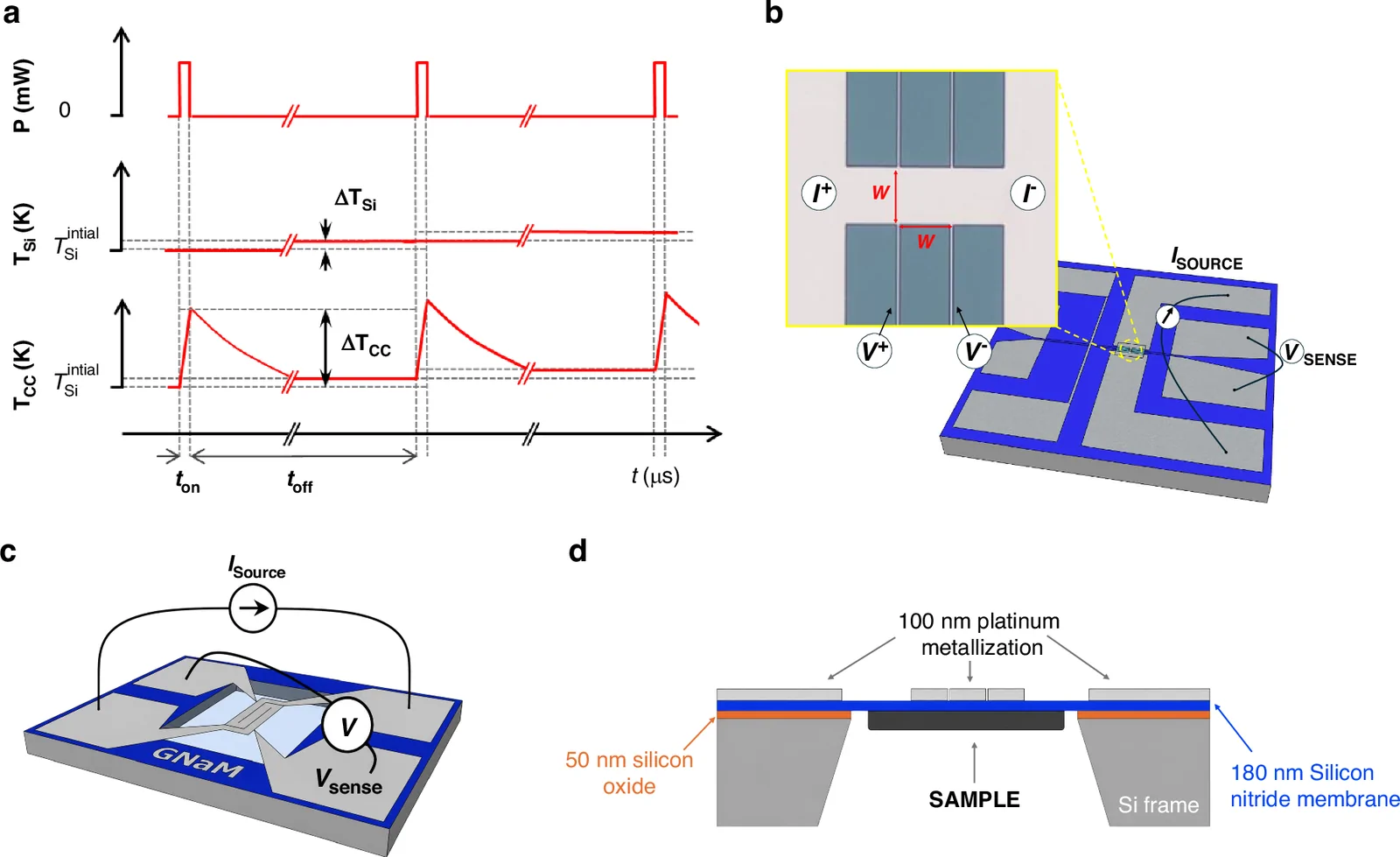
Overview and working principle of the microsecond-pulsed nanocalorimeter. **a** Microsecond current pulses are applied to the nanocalorimetric device, with a duty cycle to ensure the cooling. ${T}_{{\rm{Si}}}$ denotes the base temperature of the substrate and sample holder, while ${T}_{{\rm{CC}}}$ corresponds to the transient temperature rise of the calorimetric cell induced by each pulse. **b** Strip-shaped heater/sensor with a square sensing area of side w (w = 30 µm, or 50 µm). Inset: magnified optical microscope image of 50 × 50 µm^2^ device. **c** Meander-shaped heater/sensor with a sensing area of 1 mm². **d** Cross-sectional schematic of the calorimeter, showing the silicon nitride membrane supporting the calorimetric cell at the center, including the metallic heater/sensor and the sample positioned underneath

The microsecond-pulsed (μs-pulsed) nanocalorimetric method is a high-resolution thermal analysis technique derived from quasi-adiabatic scanning calorimetry. In this approach, the duration of the heating pulse is reduced to the microsecond range, producing a localized temperature increase of the calorimetric cell only a few Kelvin above the base temperature of the silicon frame. By applying current pulse trains with controlled duty cycles, ~50 µs ON and 100 ms OFF, the system remains near thermal equilibrium. Figure 1a shows a schematic of the train of pulses methodology. The duty cycle is chosen such that the calorimetric cell fully relaxes thermally between pulses, ensuring that each excitation starts from the same base temperature set by the substrate and sample holder, ${T}_{{\rm{Si}}}$. During each pulse, the applied current produces a rapid Joule heating of the suspended calorimetric cell, leading to a transient temperature rise ${\triangle T}_{{\rm{CC}}}$ of few K relative to ${T}_{{\rm{Si}}}$. Thus, ${T}_{{\rm{Si}}}$ represents the slowly varying base temperature of the substrate imposed by the cryostat temperature holder, while ${T}_{{\rm{CC}}}$ denotes the instantaneous temperature of the calorimetric cell induced by the microsecond pulse. During each local adiabatic heating pulse, after discarding the initial transient response, the calorimetric variables vary linearly, which allows considering time-averaged values for each variable within the ON-time window. Since the microsecond current pulse train is applied sequentially to the nanocalorimetric device, controlling the substrate temperature evolution enables additional signal averaging of the calorimetric magnitudes over repeated excitations at the same temperature, thereby enhancing sensitivity. This configuration allows heat capacity to be evaluated under quasi-static conditions, as in AC or relaxation calorimetry, while retaining the high heating rates and sensitivity characteristic of QAnC.

Figure 1b–d presents different schematic views of the nanocalorimeters employed in this study. Each device consists of a freestanding silicon nitride (Si_3_N_4_) membrane, 180 nm thick, suspended over a silicon frame. Between the silicon nitride and the silicon there is a 50 nm thermally grown SiO₂ layer, which serves as an electrical insulation layer, preventing leakage currents and shunting. At the center of the membrane, a Ti/Pt bilayer (15/100 nm) defines the metallic element that functions both as a resistive heater and as a thermometer. Three distinct heater geometries were fabricated and tested: one meander-type design with a sensing area of 1 mm², and two strip-type designs with square sensing areas of 50 × 50 µm² and 30 × 30 µm², respectively. Samples, when present, are deposited on the backside of the silicon nitride membrane. A complete geometrical layout, dimensions and optical microscope images of the fabricated nanocalorimeters are explained in the Supplementary Information.

### Principle of operation

Quantitative calorimetric measurements, irrespective of the specific architecture of the calorimetric cell, require the simultaneous determination of three key quantities: the time-dependent temperature of the cell, the electrical power input, and, under non-adiabatic conditions, the heat losses to the surroundings.

To operate the calorimeters, the local pulsed thermal excitation is achieved by injecting a controlled current I(t) through the metallic element. The resulting Joule heating induces a rapid temperature increase in the calorimetric cell, including the underlying dielectric membrane. The metallic element is designed in a four-probe configuration, allowing for localized measurement of the voltage drop V(t) at the calorimetric cell. The instantaneous power dissipated in the sensing area is calculated as ${P}_{{in}}\left(t\right)=V\left(t\right){\cdot I}\left(t\right)$, and the time-dependent resistance is given by $R\left(t\right)=V\left(t\right)/I\left(t\right)$. These quantities, combined with a prior calibration of the resistance vs temperature relationship, enable accurate reconstruction of the temperature evolution T(R(t)) and subsequent heat capacity analysis. After calibration, the platinum metallization exhibits an electrical conductivity of approximately 6.6 × 10^6 ^S/m, with a temperature coefficient of resistance (TCR) of ≈0.0026 K⁻¹. At room temperature, the measured resistance is approximately ≈20 Ω for the meander-type design and ≈1.1 Ω for the strip-type sensors, with the sheet resistance being consistent across the different strip sizes. When injecting currents on the order of tens of milliamperes, the metallic element releases sufficient power to achieve heating rates on the order of 10^5 ^K/s. Operating at such a high heating rate the nanocalorimeter behaves quasi-adiabatic.

Under high vacuum conditions, heat losses from the calorimetric cell are limited to conduction through the membrane and thermal radiation and operating at such a high heating rate the nanocalorimeter behaves quasi-adiabatic. For instance, a meander-type calorimeter requires approximately 80 mW to reach a heating rate of 10^5 ^K/s, while the effective thermal conductance at room temperature is around 4 μW/K. Consequently, only after a temperature rise of ≈200 K, the power loss term ${P}_{{losses}}$ (4 μW/K × 200 K) reaches ≈1% of the input power ${P}_{{in}}$. Therefore, for short scans, heat losses can be neglected, while for longer scans, they must be considered. Details on how to account for these losses are provided in the Supplementary Information. In quasi-adiabatic conditions, nearly all the input power contributes to the internal energy of the calorimetric cell, and the apparent heat capacity ${{Cp}}_{\beta }^{{cc}}\left(t\right)$ measured approaches the real heat capacity of the calorimetric cell ${{Cp}}_{{real}}^{{cc}}\left(t\right)$, since ${{Cp}}_{\mathrm{real}}^{{cc}}\left(t\right)$ becomes negligible:

1 $${{Cp}}_{\beta }^{{cc}}\left(t\right)=\frac{{P}_{{in}}\left(t\right)}{\beta \left(t\right)}={{Cp}}_{{real}}^{{cc}}\left(t\right)+\frac{{P}_{{losses}}\left(t\right)}{\beta \left(t\right)}$$

where $\beta \left(t\right)={dT}\left(t\right)/{dt}$ the measured heating rate.

In this framework, the measurement of a sample requires two consecutive measurements of the same calorimetric cell: first with the empty CC (denoted with superscript 0) and subsequently with the sample evaporated onto it (denoted with superscript 1).

2 $${{C}_{P}}^{{sample}}\,\left(T\right)\approx {{C}_{P}}_{\beta }^{{cc},1}\left(T\right)-{{C}_{P}}_{\beta }^{{cc},0}\left(T\right)$$

Analysis of the intrinsic noise in this measurement reveals that it is largely dominated by the numerical differentiation of digitized signals. Implementing true differential measurements significantly enhances the resolution.

**Fig. 2:**
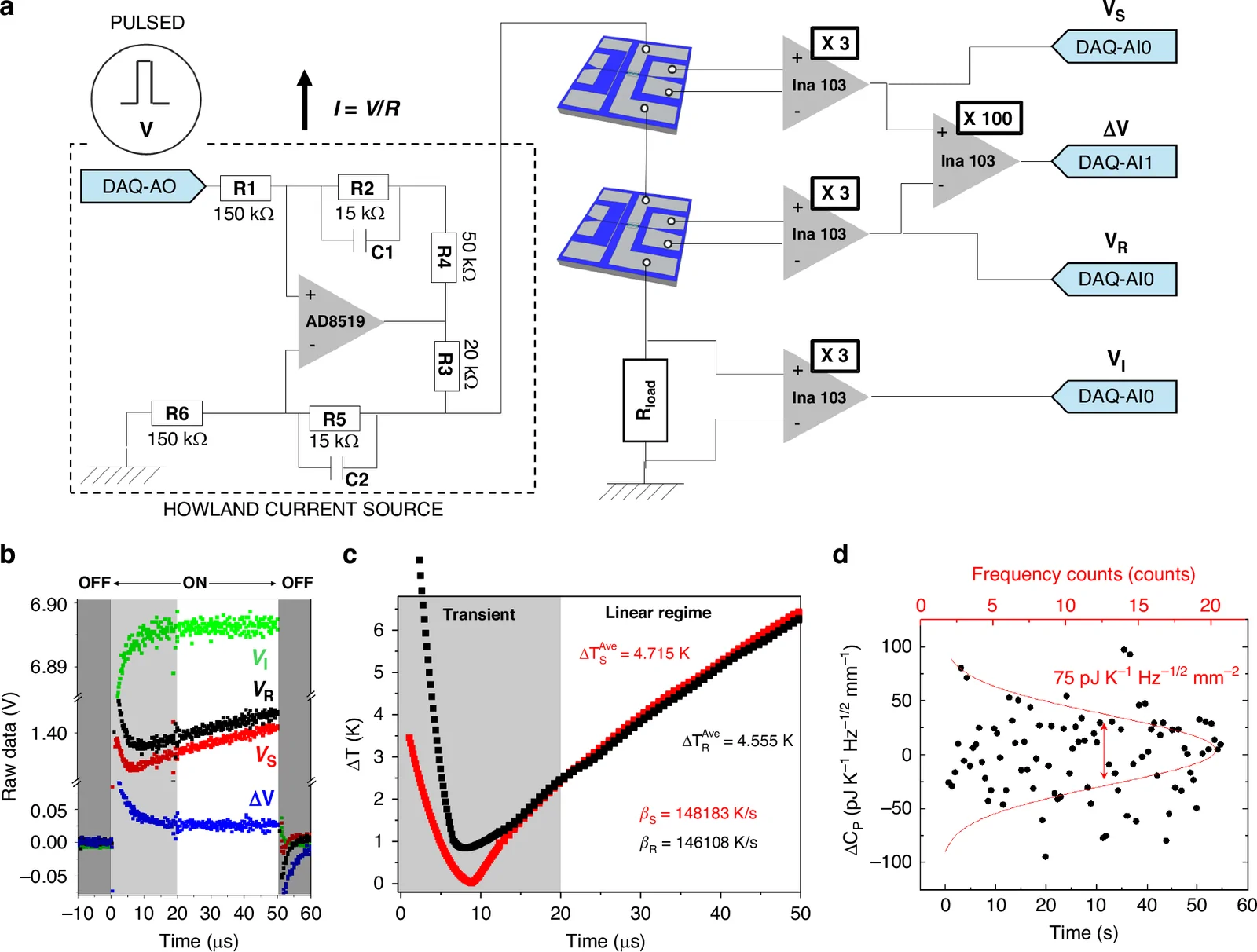
Electronic chain schematics. **a** (On the left) The ‘Howland’ Current Source based on a Op. Amplifier 8510, (On the right) an amplification bridge based on INA103, permit to measure the voltage dropped in the calorimetric devices, the differential signal and the current passing through a load resistor. **b** Raw data voltages acquired for a single pulse. **c** Temperature evolution of a couple of nanocalorimeters, showing transient zone and linear zone used to perform the evaluation of the variables. **d** Fluctuations around the mean value of the difference in heat capacity between twin nanocalorimeters (meander type) measured fixed temperature (150 K), as a function of time. The dispersion of the data determines the uncertainty of the measurement

The differential methodology can be implemented by connecting a pair of matched nanocalorimeters in series and performing the differentiation using a bridge of instrumentation amplifiers, as illustrated in Fig. 2. The differential voltage drop between the two calorimetric cells, defined as $\triangle {\rm{V}}={{\rm{V}}}_{{\rm{S}}}-{{\rm{V}}}_{{\rm{R}}}$ where cell S hosts the sample (if any) and cell R serves as the reference, can be acquired with high amplification (typically ×10 to ×1000) using an instrumentation amplifier. The difference in apparent heat capacity between the two cells can then be recalculated by incorporating the differential voltage ∆V into the general expression (the complete derivation is provided in Supplementary Information)^12,22^:

3 $${{\mathbf{\Delta }}C}_{P}\left({T}_{S}(t)\right)=\frac{I\,\Delta V}{{\beta }_{S}}-\frac{{V}_{R}}{{\beta }_{S}\,{\beta }_{R}}\,\frac{{\left(d\Delta V/{dt}\right)}_{t}}{{\left(d{R}_{S}/d{T}_{S}\right)}_{t}}+\frac{{V}_{R}\,I}{{\beta }_{S}}\left[1-\frac{{\left(d{R}_{R}/d{T}_{R}\right)}_{t}}{{\left(d{R}_{S}/d{T}_{S}\right)}_{t}}\right]$$

Finally, in a complete experiment, the measurement of the sample’s heat capacity must account for the difference between the differential heat capacities of the calorimetric cells before and after sample deposition, in order to subtract any pre-existing offset (similarly to the procedure followed in non-differential measurements):

4 $${{C}_{P}}_{{diff}}^{{sample}}({T}_{S}(t))={\Delta{C}_{P}}^{1}\,-\,{\Delta{C}_{P}}^{0}$$

If large samples (compared to the calorimetric cell addenda) are loaded into the sample calorimetric cell, the resulting heating rates when the cell is loaded, ${\beta }_{S}^{01}\left(t\right)$ relative to the idle cell ${\beta }_{S}^{0}\left(t\right)$, may vary substantially, and the baseline subtraction may require an additional correction as proposed by Efremov et al^22^. Nevertheless, in the case of ultrathin samples such as those considered in this work, and given the very short duration of the temperature excursion, the accumulated temperature differences between the sample cell in the loaded and idle experiments are typically on the order of a few millikelvin. Combined with the fact that the variation in the cell’s heat capacity is also very small, the resulting beta correction falls well below the intrinsic noise level of the measurement and can therefore be neglected.

The analysis presented here, evaluates the thermodynamic variables of the calorimetric cell as a function of time during the local heating. As mentioned above in the microsecond-pulse regime, these variables exhibit linear behavior after discarding the initial transient response, which allows assigning averaged values for each pulse.

### Instrumentation and electronics

The fast temporal dynamics of microsecond-pulsed nanocalorimetry necessitate a dedicated high-speed electronic setup. The system is built around a differential instrumentation amplifier bridge, where a pair of matched nanocalorimeters are connected in series with a precision load resistor (50 Ω). A schematic of the complete electronic chain is provided in Fig. 2a. Signal generation and acquisition are performed using a PXIe-6124 data acquisition card (National Instruments), which offers 16-bit resolution and supports simultaneous analog input/output at sampling rates up to 4 MS/s within a ± 10 V range. Synchronization and control of the pulse sequences are managed via LabVIEW software, which orchestrates the generation of current pulses by modulating the analogic output channel (DAQ-AO). The pulse parameters - including amplitude, pulse width (t_on_), and inter-pulse delay (*t*_off_) -are fully programmable, enabling precise control of the duty cycle.

To ensure stable and symmetric current delivery across varying load conditions, a Howland current source was implemented using an AD8519 operational amplifier (Fig. 2a). This voltage-to-current converter maintains a constant current through the bridge during microsecond-scale pulses and can deliver up to 67 mA in the regime used in our experiments. The Howland network was built using commercial precision 0603 SMD resistors with 1% tolerance, with the critical resistor ratios (R1/R6 and R2/R5) selected to ensure proper current-source operation. The output current is directly monitored via the voltage drop across a current-sense resistor (*R*_3_ = 20 Ω), allowing the pulse current and injected power to be reconstructed from measured quantities rather than inferred from nominal resistor values. This approach relaxes the requirements on absolute resistor matching while ensuring accurate current delivery under high slew-rate conditions. Small compensation capacitors (5 pF ± 0.25 pF, corresponding to ±5% tolerance) are included to improve stability and suppress high-frequency oscillations. The circuit design follows standard guidelines for Howland current sources, as described in Texas Instruments Application Note AN-1515 ^23^.

Voltage signatures from the calorimetric bridge are pre-amplified using an INA103 instrumentation amplifier, chosen for its fast settling time (3 μs), ultra-low input noise ($1{nV}/\sqrt{{Hz}}$ at 1 kHz), and high common-mode rejection ratio. The current delivered to each branch is monitored via the voltage drop across the load resistor (V_I_), enabling accurate reconstruction of both the instantaneous power input and the thermal response.

The initial voltage overshoot is associated with the circuit inductance responding to the high-frequency components of the current step, while the subsequent transient (~10 μs) is dominated by capacitive effects and the bandwidth-limited response of the Howland current source and instrumentation amplifiers. In optimized devices, the transient response remains below 10 μs. Considering the characteristic total resistance of the measuring circuit, the combined capacitance can be estimated at approximately 1 μF. A detailed discussion of the different impedance contributions and the device optimization required to minimize these effects is provided in the Supplementary Information.

Importantly, since the first 20 μs of the signal are discarded, the measurement is performed in the steady-state region where low-frequency components dominate and inductive and capacitive effects become negligible. This ensures that any phase difference between current and voltage does not affect the determination of thermometer resistance or power. Nevertheless, this constraint clearly imposes an upper limit on the achievable heating rates when the analyzed temperature window must remain restricted to only a few kelvin.

To conduct the experiments, the nanocalorimeters were mounted inside a custom-built liquid nitrogen immersion cryostat operating under high vacuum conditions (working pressures well below 10⁻^7^ mbar). The chamber was evacuated using a turbomolecular pump (Varian/Agilent Turbo-V 70) backed by a dry scroll pump (Agilent IDP-15). The devices were mechanically secured to an aluminum mount holder using clamps, and thermal contact was enhanced with either Apiezon N grease or silver paste. Given the high sensitivity of the measurements to base temperature fluctuations (δT₀), the temperature of the holder was actively controlled. For this purpose, the mount was equipped with a resistive heater powered by a Keithley 2400 source meter and monitored using a cryogenic Pt100 thermometer connected to a Keithley 2700 multimeter. A LabVIEW®-based control program implementing a PID algorithm was used to regulate the temperature setpoint. The use of high-precision source meters (6.5-digit resolution) enabled excellent thermal stability, achieving peak-to-peak temperature fluctuations below 1 mK. This setup allowed for both isothermal and temperature-ramp experiments across the full operational range of 80 to 450 K, a range limited not by the µs-PHnC technique but by the cryostat used and the low-temperature response of the Pt heater/sensor.

Figure 2b shows the raw voltage signals acquired during a single pulse experiment using a meander-type nanocalorimeter. The four recorded signals correspond to the sample (V_S_), reference (V_R_), current monitor (V_I_), and the amplified differential signal (100×ΔV). The acquisition is configured to capture data both before and after the pulse, enabling baseline offset correction for each channel, an essential step for accurate averaging across multiple pulses. The acquisition window includes the final part of the previous off-state (0–50 µs), the pulse-on interval (50–100 µs), and the beginning of the subsequent off-state (100–150 µs), as indicated in the figure. Only the pulse-on interval is used for calorimetric analysis. Figure 2c presents the corresponding temperature evolution of the sample and reference calorimeters. Two distinct regions are highlighted: an initial transient zone (shaded gray), dominated by the response time of the INA103 amplifier (≈3 μs), and a subsequent linear regime (white), where the temperature increases linearly with time. From this linear region, the average temperature rise after 50 μs was determined to be 4.715 K for the sample and 4.555 K for the reference. The corresponding heating rates, extracted from the slope of the linear fits, were 148183 K/s and 146108 K/s, respectively. To assess the noise density of the pulsed heating method, heat capacity was measured over 50-second intervals. Figure 2d displays the temporal fluctuations of the differential heat capacity signal (ΔCp) measured between two nominally identical nanocalorimeters at 150 K. Each black point corresponds to an individual measurement taken at a fixed acquisition rate. The dispersion of these values around zero is analyzed statistically, and the histogram of counts (top axis) is fitted by a Gaussian distribution (red curve). The standard deviation of this Gaussian represents the noise-equivalent fluctuation of ΔCp within the acquisition bandwidth. To express the resolution in a bandwidth-independent form, we normalize this standard deviation by the square root of the measurement bandwidth, yielding units of J K⁻¹ Hz⁻¹/² mm⁻². This allows comparing nanocalorimetry sensitivity across different acquisition times or instruments.

Using the experimentally determined noise-equivalent heat-capacity density of 75 pJ K⁻¹ Hz^1/2^ mm⁻², the corresponding noise floor for a reduced active area of 100 µm², representative of exfoliated two-dimensional flakes, is approximately 7.5 fJ K⁻¹ Hz^1/2^. For typical monolayer and few-layer 2D materials, whose expected heat capacities in this area range are on the order of 10–100 fJ K⁻¹ at room temperature, this translates into signal-to-noise ratios of order unity or higher with integration times of a few seconds. Longer integration times further improve the signal-to-noise ratio following the expected *t*^1/2^ scaling. Although direct calorimetric measurements on 2D materials are beyond the scope of the present work, this quantitative estimate suggests that the sensitivity achieved by µs-PHnC is sufficient for calorimetry at lateral dimensions relevant to exfoliated flakes.

### Thermal modeling and scalability of the calorimetric cell

Ideally, the temperature across the calorimetric cell should remain homogeneous during calorimetric experiments. This condition is partially met in rapid heating experiments, where power is locally dissipated in the sensing area of the heater, and lateral heat diffusion induces transient temperature gradients. In calorimetric cells with a 1 mm² sensing area, this diffusion remains moderate even on millisecond timescales, allowing quasi-adiabatic operation. However, with the implementation of the microsecond-pulsed nanocalorimetry (µs-PHnC) method, the temperature profile improves substantially.

Finite element modeling of devices with silicon nitride membranes of 180 nm thick and 150 nm of Pt metalization (details provided in the Supplementary Information) reveals that under 50 µs pulses at heating rates of 100 kK/s, approximately two-thirds of the sensing area exhibit temperature differences below 2.5 K. In contrast, for 1 ms pulses, temperature variations can reach up to 20 K across two-thirds of the calorimetric cell. Although the thermal inhomogeneity inherent to the meander-type design can be mitigated by integrating additional metallic spreaders within the sensing area, this approach introduces a significant increase in heat capacity addenda, thereby compromising the overall sensitivity of the calorimetric cell ^20^.

**Fig. 3:**
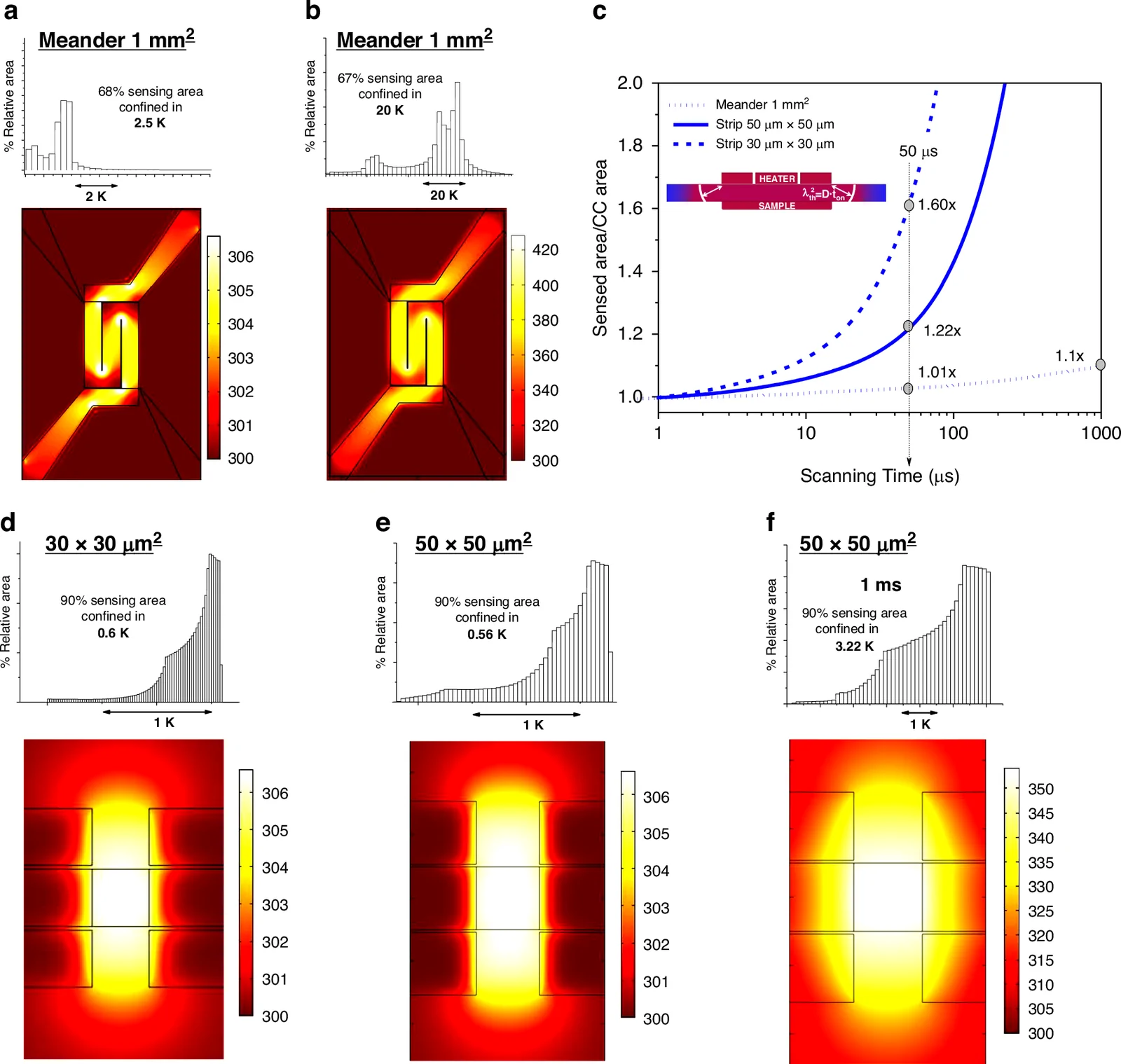
Thermal homogeneity and scalability of the calorimetric cell. Temperature maps of a meander like calorimeter heated at 10^5^K/s at times of **a** 50 μs and **b** 1 ms. The histograms show the 2D temperature dispersion in the calorimetric cell: showing that 2/3 of surface area is within 2.5 K after 50 µs from the start of the pulse while the temperature inhomogeneity degrades to 20 K after 1 ms. **c** Comparison of the sensed area normalized to the calorimetric cell (CC) area versus scanning time for three thermal sensing configurations: Meander (1 mm²), Strip (50 μm × 50 μm), and Strip (30 μm × 30 μm). **d**–**f** Show Simulated temperature maps for strip-type nanocalorimeters with sensing areas of 30 × 30 µm² and 50 × 50 µm². At 50 µs, 90% of the sensed area remains within a temperature range of 0.6 K and 0.56 K, respectively, indicating high thermal homogeneity. For the 50 × 50 µm² device at 1 ms, the temperature spread increases to 3.22 K, highlighting the impact of thermal diffusion over time on spatial uniformity

Figure 3a, b display the simulated temperature maps and corresponding histograms for a meander-type calorimetric cell (1 mm²) at 50 µs and 1 ms, respectively.

To assess how spatial temperature degradation under fast heating affects heat capacity measurements and limits calorimeter miniaturization, we used finite element modeling to show that the sensed heat capacity increases with thermal diffusion length, which scales with the square root of membrane diffusivity and time. The simulations reveal deviations in the measured heat capacity, and thus in the effective sensed area or heat capacity addenda, from the intrinsic values of the calorimetric cell. Figure 3c shows the normalized sensed area over time for different device geometries, based on comparisons between the heat capacity measured at a heating rate of 100 kK/s and the intrinsic heat capacity at the corresponding temperature, The inset schematically illustrates the thermal diffusion length λ, given by λ² = Dₜₕ·time, where *D*ₜₕ is the thermal diffusivity of the membrane. The analysis includes meander-type devices with 1 mm² sensing areas and strip-type nanocalorimeters with 50 × 50 µm² and 30 × 30 µm² sensing areas.

For meander-type devices with a ≈ 1 mm² sensing area, the sensed area increases by only ~1% after 50 µs (within the resolution limit) and reaches ≈10% after 1 ms. This moderate expansion follows a time dependence of t^1/2^ and remains manageable even at millisecond timescales. In quasi-adiabatic nanocalorimetry (QAnC), this effect can be mitigated by using differential measurements and by confining the sample to the original calorimetric cell with a shadow mask.

In contrast, for miniaturized strip-type devices with sensing areas of 50 × 50 µm² and 30 × 30 µm², the influence of thermal diffusion becomes significantly more pronounced. After 50 µs, the sensed area increases by approximately 22% and 60%, respectively. For these devices, the sensed area doubles the original calorimetric cell area after just 220 µs (50 × 50 µm²) and 80 µs (30 × 30 µm²), clearly demonstrating that long-duration pulses are unsuitable for small-scale designs. The rapid expansion of the sensed area severely limits sensitivity and imposes a fundamental constraint on the scalability of calorimetric cells and their applicability in QAnC.

The µs-PHnC method, operating in the tens of microseconds regime, effectively minimizes this diffusion-driven expansion. By maintaining a reduced sensed area, it also limits the heat capacity addenda, a key factor in enhancing sensitivity. This makes µs-PHnC particularly well-suited for high-resolution calorimetry in ultrasmall samples.

In addition to the broad variation in lateral device dimensions explored in this work, further miniaturization into the sub-micron regime introduces additional constraints that must be considered for future implementations. At these length scales, the thermal diffusion length during a 50 µs pulse becomes comparable to the footprint of the device, making thermal uniformity increasingly sensitive to both the scan speed and the exact geometry of the heater–thermometer strip. Accurate sample delineation also becomes non-trivial at sub-micron scales: lithographically defined shadow masks or well-aligned deposition windows are required to prevent overspread of material beyond the active region, which would otherwise dominate the heat-capacity signal.

Figure 3d–f)presents simulated temperature profiles for strip-type devices with sensing areas of 30 × 30 µm² and 50 × 50 µm². At 50 µs, the heated region closely matches the heater footprint, which represents the ideal scenario. However, a narrow region surrounding the heater also becomes heated due to thermal diffusion, extending a few micrometers beyond the active area. This effect is more noticeable in the smaller device and becomes increasingly significant at longer pulse durations, as shown in Fig. 3c.

The improvement in temperature homogeneity is evident. For the 50 × 50 µm² calorimeter, 90 percent of the sensed area falls within a temperature range of 0.56 K at 50 μs, as shown in the histogram. As expected, increasing the pulse duration or reducing the device size leads to greater temperature dispersion. For example, at 50 μs, the 30 × 30 µm² device shows a temperature spread of 0.6 K, while the 50 × 50 µm² device reaches 3.22 kelvin at durations of 1 ms. These results highlight the trade-off between temporal resolution and spatial uniformity in miniaturized calorimetric designs.

## Results and discussion

To demonstrate the capabilities of the microsecond-pulsed heating nanocalorimetry (µs-PHnC) technique and its scalability to reduced sensing areas, we fabricated three nanocalorimetric devices: one with a meander-type geometry (1 mm^2^) and two with strip-type geometries featuring calorimetric cell sizes of 30 × 30 µm² and 50 × 50 µm², respectively. Thin films of cobalt monoxide (CoO) were deposited onto the backside of the calorimetric membranes using electron beam evaporation^24^. The antiferromagnetic transition of CoO was selected as a benchmark due to its continuous nature and absence of latent heat, making it an ideal candidate for evaluating the resolution of the technique.

Prior to sample deposition, as in quasi-adiabatic nanocalorimetry (QAnC), the baseline heat capacity difference between the calorimetric cells was measured using both non-differential and differential configurations. During Cp(T) measurements, the holder temperature was continuously scanned from 100 K to 350 K at a slow and controlled rate of 0.016 K s⁻¹ (1 K min⁻¹), ensuring excellent thermal stability throughout the entire range. Each data point was obtained by applying microsecond heating pulses with a duration of 50 µs, corresponding to an effective heating rate of approximately 1.2 × 10⁵ K s⁻¹. To avoid artefacts associated with the electrical transient at the onset of the pulse, the first 10 µs of the response were excluded from the analysis. At each temperature, 10 pulses per second were applied and averaged, yielding a single Cp value with improved signal-to-noise ratio while preserving fine temperature resolution. One of the main limitations of µs-PHnC compared to QAnC is the longer acquisition time required to achieve high-resolution measurements. This extended duration increases the susceptibility of the calorimetric cell to contamination from residual water or other adsorbates. To mitigate this, a preconditioning step was implemented before each measurement, consisting of either a rapid temperature ramp to 400 K or a flash heating cycle to 600 K. These procedures effectively desorb unwanted species from the calorimetric cell. When this protocol is followed, the measurements exhibit excellent reproducibility.

**Fig. 4:**
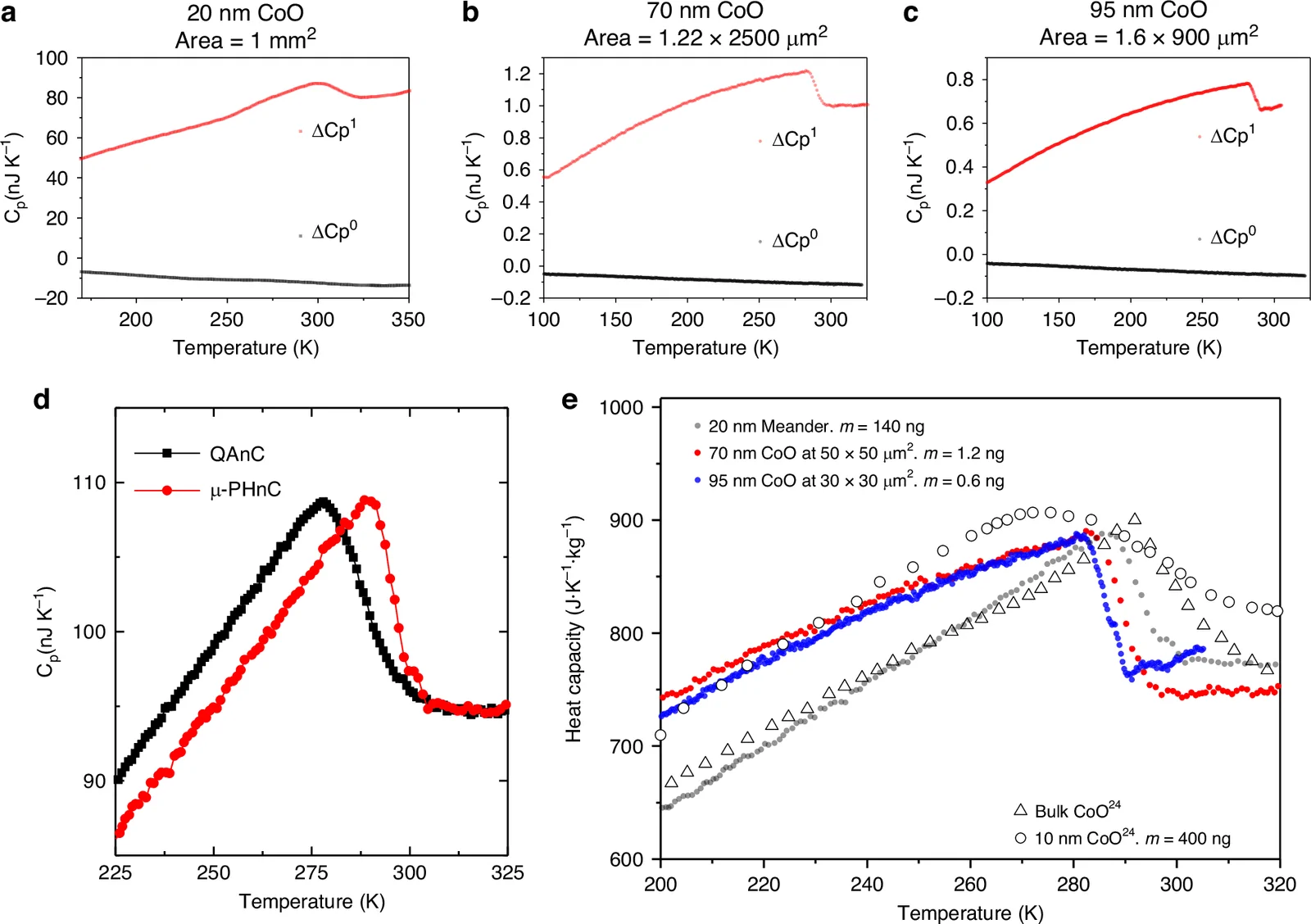
Differential heat capacity (ΔCp) measurements of CoO thin films with varying sensing areas and nominal thicknesses. The three panels correspond to: **a** A 20 nm CoO film measured on a meander-type calorimeter (1 mm² sensing area), **b** A 70 nm CoO film on a 50 × 50 µm² strip-type device, and **c** A 95 nm CoO film on a 30 × 30 µm² strip-type device. In each case, ΔCp⁰ (black) represents the baseline differential signal prior to sample deposition, and ΔCp¹ (red) corresponds to the signal after sample deposition. **d** Heat capacity of the same sample in meander device, 20 nm CoO, measured by QAnC (black squares), and μ-PHnC (red circles). **e** Specific heat capacity of CoO thin films measured using µs-PHnC across different calorimetric cell geometries

A key advantage of the µs-PHnC technique is that it eliminates the need for a shadow mask to confine the sample. The effective analysis area is inherently defined by the heater geometry and the thermal diffusion length corresponding to the pulse duration. In this study, a 20 nm CoO film was deposited on the meander-type device with the aid of a shadow mask that limits the sample to the active area of the calorimetric cell, while 95 nm and 70 nm CoO films were deposited shadowless on the 30 × 30 µm² and 50 × 50 µm² strip-type devices, respectively. Film thicknesses were estimated using a QCM and calibrated via profilometry, with an associated uncertainty of approximately 10%. Figure 4a–c presents the differential heat capacity curves before and after CoO deposition for each of the calorimetric cell designs. The data clearly illustrate the thermal response of the system and the effectiveness of µs-PHnC in capturing the heat capacity signature of the CoO thin films across different device geometries.

The improvement in temperature uniformity achieved with microsecond-pulsed heating nanocalorimetry (µs-PHnC), shown in Fig. 3, is experimentally validated by analyzing the width of the calorimetric anomaly associated with the Néel transition in CoO thin films. For samples fabricated via physical vapor deposition (PVD), the grain size is expected to lie within the nanometer regime^24^. In CoO, this antiferromagnetic-to-paramagnetic transition appears as a lambda-type anomaly in the heat capacity curve. While this transition is sharp in bulk materials, thin films exhibit a broadened transition due to finite-size effects, particularly when the grain size approaches the correlation length of the superexchange interaction. Figure 4d presents a comparative analysis of the heat capacity signal for a CoO film with a nominal thickness of ~20 nm (as determined by quartz crystal microbalance, QCM), measured using both quasi-adiabatic nanocalorimetry and µs-PHnC. In the quasi-adiabatic regime, the transition spans approximately 20 K, consistent with the thermal gradients predicted for millisecond-scale heating. In contrast, µs-PHnC measurements reveal a significantly narrower transition (≈5 K), which lies above the modeled temperature dispersion, thereby offering a more faithful representation of the intrinsic rounding induced by the nanocrystalline structure.

The subtraction of the differential heat capacity values before and after sample deposition yields the net heat capacity of the CoO thin film. To calculate the specific heat, we consider the effective analysis areas obtained from finite element modeling, the nominal thicknesses estimated via QCM calibration, and an assumed density of 6450 kg m⁻³, corresponding to the bulk value for CoO. It is worth noting, however, that thin films deposited by physical vapor deposition (PVD) may exhibit slightly lower densities due to microstructural effects. Figure 4e presents the calculated specific heat values for each device configuration. Red circles correspond to a 70 nm CoO film measured on a 50 × 50 µm² strip-type device; blue circles represent a 95 nm CoO film on a 30 × 30 µm² strip type device; and black circles correspond to 20 nm CoO films measured on meander-type devices. For comparison, literature data from Tang et al^25^. are included: open triangles for 10 nm CoO and open circles for bulk CoO. The sharp lambda-type anomaly near 290 K reflects the Néel transition, with the µs-PHnC technique capturing the transition with high resolution across all device configurations. The corresponding sample masses for each measurement are indicated in the legend. It is worth to highlight that strip-type µs-PHnC provides a ~ 10^3^- fold reduction in sample mass relative to previous study^25^, while maintaining excellent signal-to-noise. The technique’s high temporal resolution and spatially confined heating minimize instrumental broadening, yielding a sharply defined magnetic transition. The same attributes can be further exploited to resolve with unprecedented resolution the subtle transition rounding commonly attributed to finite-size effects when downscaling to thicknesses of only a few nm. Crucially, µs-PHnC resolves phase transitions in sub-nanogram specimens, paving the way for measurements on single-monolayer 2D materials and other ultra-small solids.

To place these results in a broader context, Table 1 compares µs-PHnC with the main chip-based nanocalorimetric approaches, namely relaxation, AC, and quasi-adiabatic nanocalorimetry. The comparison is focused on the most representative and significant implementations among those operating in the intermediate temperature range relevant to the present work, approximately 30–300 K. The table summarizes the corresponding operating regimes, signal-enhancement strategies, temperature homogeneity, accessible temperature range, addenda, heat-capacity resolution, lateral sensing area, and characteristic timescales. This overview highlights that µs-PHnC occupies a distinct regime, combining the high heating rates of fast scanning methods with quasi-isothermal operation and signal averaging, while extending nanocalorimetry to micrometer-scale.

**Table 1:** Comparison of representative electrically actuated chip-based nanocalorimetric methods

Parameter\nanocalorimetric method		Relaxation nanocalorimetry	AC-nanocalorimetry	Quasi adiabatic nanocalorimetry	μs-pulsed heating
Representative works		Bachmann et al.^2^ ; Denlinger et al.^6^ ; Queen et al.^26^ ; Wickey et al. ^27^	Corbino^8^; Sullivan and Seidel^10^; Bourgeois et al.^9^ ; Shoifet et al.^14^ ; Huth et al.^28^ ; Xiao et al.^29^; Ftouni et al.^30^ ; Le et al. ^31^.	Allen et al.^4^ ; Rodríguez-Viejo et al.^12^ ; Zhuravlev and Schick^13^; Efremov et al.^19,22^ ; Zhang et al.^21^. ; Franke et al.^32^.	This work
Dynamics regime		Quasi-Isothermal; Small excitation measuring thermal relaxation times.	Quasi-Isothermal; Periodic excitation reaching AC steady state.	Scanning; Large excitations promoting ultrafast heatings over large T scans	Quasi-Isothermal; Large excitations promoting ultrafast local heatings.
Enhancement of the calorimetric signal	Increasing heating rates	Limited by thermal relaxation time; larger ΔT reduces T uniformity and introduces offsets.	Limited by thermal relaxation time; larger ΔT reduces T uniformity and introduces offsets.	High current excitation; to achieve high scan rate and signal amplitudes.	High current excitation; to achieve high scan rate and signal amplitudes.
	Signal treatment	Bridge amplification; Repeated transient averaging; Differential measure;	Lock-in amplification; Amplitud and Phase sensitive detection of thermal oscillation.	Bridge amplification; Differential measure; Scan averaging for reversible processes;	Bridge amplification; Differential measure; Scan averaging for reversible processes;
Excitation temperature ΔT		From few mK to K	From few mK to K	From 10 K to 10^3^ k	From 0.1 K to few K
Temperature homogeneity in the calorimetric cell		Very Good; Inhomogeneities below the excitation temperature	Very Good; Inhomogeneities below the excitation temperatura.	T inhomogeneities from few K to 50 K depending on heating rates and scan length.	Very Good; Inhomogeneities below the excitation temperature.
Temperature range		From few mK to 800 K	From few mK to 10^3 ^K	From 30 to 10^3 ^K	From 30 to 400 K
First order transitions (enthalpy/kinetics)		Limited; Not ideal choice.	Limited; Model dependent in adiabatic regime ^33^.	Very well adapted; Widely used in kinetic studies.	Limited; Not ideal choice.
Continuous transitions (second order and λ-type)		Very well adapted	Very well adapted	Very well adapted	Very well adapted
Compatibility with external fields or variables (Magnetic, Electric, Pressure…)		Good; Operating quasi- isothermal	Very good; Operating quasi-isothermal. Possibility to coupled with oscillating fields.	Limited; Only over large temperature scans.	Good; Operating quasi- isothermal
For decices operating at room temperature	Cp Addenda	~200 nJ K^−1 27^ ~4 nJ K^−1^ ^27^	~10 nJ K^−1 29^ ~ 1800 nJ K^−1 30^ ~ 100 nJ K^−1^ ^30,31^	~100–1000 nJ K^−1^ ^21,24,32^	~1–1000 nJ K^−1^
	Cp resolution	~10 nJ K^−1 2r7^ ~ 0.1 nJ K^−1 28^	~50pJ K^−1 29^ ~ 5–10 nJ K^−1 30^ ~ 1–2 nJ K^−1^ ^30,31^	30pJ/K^21^	~100 fJ/K–75 pJ/K
	Area of analysis	~1×1 mm^2^ ^26^. ~100×100 μm^2^ ^27^, Linked to the thermal diffusion length; Reduction requires complex designs.	~50 × 100 μm^2^ ^28^ –0.45 mm² ^30,31^ ~ 2.5 mm² ^29^ Linked to the thermal diffusion length.	~mm^2^ ^21,24,32^ Lateral diffusion length along large temperature scans spread sensing area limiting miniaturization.	~30 × 30 μm^2^ to mm^2^ Linked to the thermal diffusion length from few tens of μm^2^.
	Cp resolution per area	~10 nJ K^−1^ mm^−2^ ^26,27^	~2–5 nJ K^−1^ mm^−2^ ^29^–5 nJ K^−1^ mm^−2^ ^30,31^ ~10 nJ K^−1^ mm^−2^ ^28^	~30 pJ K^−1^ mm^−2^ ^21^	~75 pJ K^−1^ mm^−2^
	Heating rates	10^2^–10^3^ K/s	10^2^–10^3^ K/s	10^4^–10^6^ K/s	~10^5^ K/s
	Characteristic time	10^−1^–10^−2^ s	1–10^−3^ s	~ms	tens of μs

The table summarizes the operating regime, signal-enhancement strategy, temperature homogeneity, accessible temperature range, addenda, heat-capacity resolution, lateral sensing area, heating-rate range, and characteristic timescale for relaxation, AC, quasi-adiabatic, and µs-pulsed nanocalorimetry. The values reported correspond to representative and significant implementations from the literature, selected among methods operating in the intermediate temperature range

## Conclusions

We have developed and validated a novel microsecond-pulsed heating nanocalorimetry (µs-PHnC) technique that enables high-resolution, quasi-isothermal heat capacity measurements in ultrathin films and low-dimensional materials, allowing thermal characterization in nanosystems that was previously beyond reach. This method builds upon the principles of quasi-adiabatic nanocalorimetry (QAnC) while overcoming its limitations in spatial resolution and scalability. By reducing the pulse duration to the microsecond regime (typically 50 µs), µs-PHnC minimizes lateral thermal diffusion, preserving temperature homogeneity and enabling accurate measurements even in calorimetric cells with sensing areas as small as 30 × 30 µm².

The technique was implemented using custom-fabricated nanocalorimeters with meander- and strip-type geometries, and its performance was benchmarked using the antiferromagnetic transition in CoO thin films. The results demonstrate that µs-PHnC captures the lambda-type anomaly with significantly improved sharpness compared to QAnC, reducing the transition width from ≈20 K to ≈5 K. Finite element modeling confirms that the sensed area remains confined to the heater footprint during short pulses, which is critical for maintaining sensitivity and minimizing heat capacity addenda. Consequently, there is little benefit in reducing the strip-type calorimeter size below ≈30 × 30 µm², since the thermal diffusion length linked to pulse duration in that range and prevents further reduction of the effective sensing area.

The µs-PHnC method also offers practical advantages: it eliminates the need for shadow masks, simplifies sample deposition, and supports integration with external stimuli under quasi-static conditions. Because the system remains near thermal equilibrium between pulses, the technique is conceptually compatible with coupling to slowly varying external fields (such as magnetic, electric, or optical stimuli) enabling the study of field-dependent thermodynamic responses with high temporal and thermal resolution.

The differential measurement scheme, combined with high-speed electronics and robust thermal modeling, ensures excellent reproducibility and noise performance, with a noise density as low as 75 pJ K^−1^ √Hz mm^−2^. The newly designed calorimeters further expand the capabilities of nanocalorimetry by allowing heat capacity measurements of materials with extremely small surface areas and heat capacity addenda below 10^−9^ J/K. This advancement paves the way for high-resolution heat capacity measurements of materials that were previously inaccessible. While traditional high-resolution Cp measurements were limited to thin-film materials, this technique now extends the capability to nanostructured and 2D materials with significantly smaller analysis areas. This represents a critical step forward in the study of thermodynamic properties of low-dimensional and nanostructured systems, broadening the scope of calorimetric applications in material science.

## Supplementary information

Supplemental Materials
