# Supplementary material for: Microsecond-pulsed nanocalorimetry: a scalable approach for ultrasensitive heat capacity measurements
Source: Microsyst Nanoeng. 2026 Sep 20;12:323. doi: 10.1038/s41378-026-01426-7 (PMC13590478; doi:10.1038/s41378-026-01426-7)
Supplement: Supplementary file 1 [file 41378_2026_1426_MOESM1_ESM.docx]

**Supplementary Information**

Microsecond-Pulsed Nanocalorimetry: A Scalable Approach for Ultrasensitive Heat Capacity Measurements

Hugo Gómez-Torres^1,2†^, Manel Molina-Ruiz^2†^^, Simone Privitera^1,2^, Enric Menéndez^2^, Llibertat Abad^3^, Jordi Sort^2,1,4^, Olivier Bourgeois^5^, Javier Rodriguez-Viejo^1,2^, Aitor Lopeandia^1,2*^

^1^ Catalan Institute of Nanoscience and Nanotechnology (ICN2), CSIC and BIST, E-08193 Bellaterra, Spain.

^2^ Departament de Física, Universitat Autònoma de Barcelona, E-08193 Bellaterra, Spain.

^3^ Institut de Microelectrònica de Barcelona (IMB-CNM-CSIC). Campus de la UAB, E-08193, Cerdanyola del Vallès (Barcelona), Spain.

^4^ Institució Catalana de Recerca i Estudis Avançats (ICREA), Pg. Lluís Companys 23, E-08010 Barcelona, Spain.

^5^ Univ. Grenoble Alpes, Institut Néel - CNRS UPR2940, 25 rue des Martyrs, BP 166, 38042, Grenoble Cedex 9, France

^†^ *Both authors contributed equally to this work*

^^^ Current address: Department of Physics, University of California, Berkeley, California 94720, USA

*Corresponding author e-mail[: aitor.lopeandia@uab.cat](mailto::%20aitor.lopeandia@uab.cat)

**S1.- Cryogenic Experimental setup**.

Calorimetric measurements of ultralow-mass samples place stringent requirements on the experimental environment. In particular, heat-capacity measurements must be performed under high-vacuum conditions in order to suppress parasitic heat losses through gas conduction and convection, which would otherwise introduce uncontrolled heat exchange between the calorimetric device and the surrounding medium. In addition, precise and stable temperature control is essential, with a target stability on the order of 1 mK, together with the capability to operate over a broad temperature range. Figure S1 shows the custom-built immersion cryostat developed for nanocalorimetric measurements of ultralow-mass samples.

The immersion cryostat consists of a stainless-steel tube approximately 50 cm in length, terminated by a massive copper end block that acts both as the lower closure of the system and as the access point for sample mounting. The two elements are joined by a high-elasticity silver-based solder, chosen to accommodate the differential thermal contraction of stainless steel and copper during cryogenic cooling and thus maintain mechanical integrity over repeated thermal cycles. The lower opening can be accessed for sample mounting and then sealed with a mechanical indium seal. This configuration provides both vacuum tightness and efficient thermal coupling to the liquid-nitrogen bath. The copper block is in direct thermal contact with the external liquid nitrogen while simultaneously defining the high-vacuum environment inside the cryostat, allowing the sample holder to be cooled close to 77 K under high-vacuum conditions.

In this system, all electrical leads connected to the sample are first thermalized to the massive copper block at the base temperature in order to minimize thermoelectric drifts and spurious thermal offsets. The nanocalorimetric chips are mounted on a custom-designed PCB socket fabricated on an aluminum-based substrate. They are attached using silver paint to ensure good thermal coupling, while the socket geometry enables wire bonding in close proximity to the devices. The PCB is mechanically secured with a screw and thermally anchored, using Apiezon grease, to a copper platform whose temperature can be controlled over the full measurement range. This platform is linked to the massive copper thermal reservoir by a thin stainless-steel tube that serves as a controlled thermal link. The platform is equipped with a cryogenic Pt100 resistance thermometer and a Kapton resistive heater, both driven by a proportional–integral–derivative (PID) control loop implemented with high-precision sourcemeters and automated through LabVIEW.

To minimize surface contamination, especially from ambient moisture adsorbed during each opening of the system for sample mounting, a preconditioning protocol is applied before every low-temperature measurement. The protocol consists of heating the sample holder to elevated temperatures (up to 400 K) or applying short local flash-heating cycles using the nanocalorimeters, in order to desorb residual gases and weakly bound adsorbates from the membrane-based calorimetric cells. This treatment is essential to ensure a high level of reproducibility in the calorimetric measurements.

**Figure S1. Immersion cryostat and experimental setup for nanocalorimetry.** (a) Schematic view of the immersion cryostat, consisting of a stainless-steel tube terminated by a massive copper end block that provides the lower access for sample mounting. (b) Exploded schematic of the lower part of the cryostat, showing the copper end block, the internal sample-holder assembly, and the screw-mounted contact structure. (c) Photograph of the assembled experimental probe, including the custom PCB socket holding the twin nanocalorimetric chips, the electrical wiring, and the indium-sealed lower closure. The copper end block is placed in direct thermal contact with the liquid-nitrogen bath, while the interior of the probe is maintained under high-vacuum conditions.

**S2.- Finite Element Modeling of the Nanocalorimeters**.

Finite element method (FEM) simulations are performed using COMSOL Multiphysics^®^ using PDE modules to combine “heat transfers in solids’ with “electrical conductive media”. The thin film nature of the nanocalorimetric device membrane guaranties that vertical temperature gradients can be neglected permitting to mathematically reduce the problem to a 2D configuration, considering a constant $d_{z}$ thickness along the structure. The simplified two-dimensional model should consider the equivalent physical properties of the stack of materials for each physical domain, referenced to apparent thickness.

Under this approximation heat transfer equation can be rewritten as,

$$d_{z}\rho C_{p}\frac{\partial T}{\partial t}-d_{z}\nabla k\nabla T=d_{z}Q_{e}+q_{o}$$

where $\rho C_{p}$ is the density times the heat capacity, $T$ is the temperature, $k$ is the thermal conductivity, $Q_{e}$ is the electromagnetic Joule heating ($Q_{e}=J\cdot E$) and $q_{0}$ the out of plane heat radiative component,

$$q_{0}=\epsilon_{u}\sigma\left( T_{ext,u}^{4}-T^{4} \right)+\epsilon_{d}\sigma\left( T_{ext,d}^{4}-T^{4} \right)$$

being $\epsilon_{u}$ and $\epsilon_{d}$, the up and down emissivity of the surface, $T_{ext,u}$ and $T_{ext,d}$ the external reference temperatures in both sides of the surface and $\sigma$ is the Stephan Boltzmann constant. Convective heat losses components are not considered since nanocalorimeters normally operate at high vacuum pressures and thus can be neglected.

Under 2D approximation Maxwell equations can also be implemented considering the $d_{z}$ dimension on the evaluation of the extensive values of the electrical conductance.

For the different designs, the planar geometries of the nanocalorimeters under study were defined using the CAD tools available in COMSOL Multiphysics^®^. As an example, the resulting layouts and mesh for the strip heater with sensing area of 50μm x 50μm are shown in Figure S2. For each domain, the effective physical properties of the stacked materials were considered. In particular, the thermal conductivity $k$ for each region was calculated as described below:

$$k=\sum_{i=0}^{n} k_{i}\cdot\left( \frac{d_{i}}{d_{z}} \right)$$

where $k_{i}$ is the thermal conductivity of the *i*-th material layer, $d_{i}$is its actual thickness, and $d_{z}$ is the apparent total thickness of the region in the model. This approach ensures that the contribution of each material is properly weighted according to its relative thickness within the stack.

**Figure S2. Layout and mesh of a strip-type calorimetric cell.** Representative layout and finite element mesh of a calorimetric device with a 50 μm × 50 μm sensing area. The cross-sectional view highlights two distinct regions composed of stacked platinum and silicon nitride layers.

When defining the heat capacity of the regions, we considered that the density is also defined common to the region and material, and should be weighed as follows:

$$\rho C_{p}=\sum_{i=0}^{n} \rho_{i}C_{p,i}\cdot\left( \frac{d_{i}}{d_{z}} \right)$$

Material properties and their temperature dependence were obtained from literature and from previous experiments conducted in our laboratory. The thermal conductivity of the silicon nitride layer, including its temperature dependence, was determined from measurements using the Völklein method ^1^. The thermal conductivity of the platinum layer was derived from the electrical resistivity of the heater strip, applying the Wiedemann–Franz law.

Figure S3 shows the electrical conductivity values used in the modelling. These values were obtained as averages from the resistivity extracted through resistance–temperature calibration of four calorimetric device designs (a meander-type and three strip geometries), all featuring 150 nm-thick platinum metallization. The resulting conductivity values enabled accurate reproduction of the experimental resistance across the different heater geometries in the simulations.

**Figure S3. Electrical conductivity derived from heater resistance calibration:** Electrical conductivity was extracted from the temperature-dependent calibration curves of the heater resistance, taking into account the geometrical parameters. For devices with a 150 nm-thick platinum metallization, the extracted conductivity values accurately reproduce the experimental resistance across different heater geometries in the simulations.

To determine the heat capacity values, we employed the Debye model, using as an upper bound the Dulong–Petit limit: 1050 J·K^-1^·kg^-1^ for silicon nitride and 135 J·K^-1^·kg^-1^ for platinum. The temperature dependence was introduced by adjusting the Debye temperature, set at 225 K for the platinum thin film and 550 K for the silicon nitride membrane. These adjusted values are lower than those reported for bulk materials, which is consistent with the expected reduction in Debye temperature due to finite-size effects in the thin films. Since the variation of the mass density is very small in the temperature range of the experiments, we considered constant values of 3100 Kg·m^-3^ and 21400 Kg·m^-3^, for silicon nitride and platinum, respectively.

Among the various parameters, the actual thickness $d_{i}$ of the constituent layers carries the greatest uncertainty and was therefore treated as an adjustable parameter in the modelling. Using the pulsed heating technique, we experimentally measured the heat capacity of the empty calorimetric cell, and applying the same procedure to the simulated data we can compare the results. In this context, increasing the thickness of either the silicon nitride layer $d_{SiNx}$ or the platinum layer $d_{Pt}$, significantly impacts the total heat capacity of the system.

Fortunately, the transient temperature response to a current pulse allows us to decouple these two contributions and refine the parameter estimation. Given that platinum has a thermal conductivity approximately 25 times higher than that of silicon nitride, even small variations in the estimated thickness noticeably affect the thermal transient. A good agreement between experiment and simulation was achieved by assuming a platinum thickness consistent with the nominal range (150 nm) and a silicon nitride thickness of approximately 173 nm, close to the nominal value of 180nm. Figure S4 illustrates both the agreement between the simulated and measured heat capacity of the empty calorimetric cell, and the fidelity of the model in reproducing the transient thermal response under a current pulse.

Although radiative losses can be considered negligible in microsecond-scale pulsed experiments, since the temperature increase is only a few kelvin above the base temperature, we included emissivity values obtained from previous nanocalorimetric measurements. The upper emissivity $\epsilon_{u}$ was set to approximately 0.1 for domains capped with platinum, and around 0.54 for domains exposing bare silicon nitride. At the experimental temperatures, the dominant infrared wavelengths have a penetration depth greater than the characteristic thickness of the silicon nitride membranes. As a result, the effective emissivity is expected to be lower than the bulk value (≈ 0.9), where deeper atomic layers also contribute to radiation. For the emissivity on the dorsal side $\epsilon_{d}$, we also considered a value of 0.54 for the bare silicon nitride membrane. When the membrane is covered with platinum on the upper side, we accounted for the additional contribution of the platinum layer, due to the partial transparency of the silicon nitride, resulting in a total effective emissivity of approximately 0.64.

**Figure S4. Adjustment of material parameters via pulsed heating.** (a) Heat capacity measurement of an empty calorimetric cell (50 μm × 50 μm) using a pulsed heating technique, compared with finite element method (FEM) simulations incorporating adjusted material parameters. (d) The temperature evolution under a constant 17.5 mA current pulse is shown, with FEM simulations considering varying platinum metallization thicknesses to assess their influence on thermal response.

FEM simulations allow us to determine temperature profiles at specific time points. In addition to this, we used FEM simulations to reproduce the experimental response observed when measuring a thin film of cobalt monoxide (CoO). Cobalt monoxide exhibits a characteristic lambda-shaped anomaly in its heat capacity associated with the antiferromagnetic transition near 290 K. Given that antiferromagnetic superexchange interactions are typically sensitive to finite-size effects, we considered heat capacity data reported in the literature for nanometric CoO samples ^2^.

Figure S5 shows the heat capacity curves for bulk CoO and for a sample with 10 nm grain size, both of which were implemented as material models in COMSOL Multiphysics ®. For the in-plane thermal conductivity, we used a value of 0.9 W·m⁻¹·K⁻¹, measured on a 50 nm-thick film using the Volklein technique. The density was set to the tabulated bulk value of 6450 kg·m⁻³ at room temperature. However, since the samples are polycrystalline thin films deposited by electron beam evaporation, it is expected that the actual density may be slightly lower.

**Figure S5. Specific heat capacity of CoO in bulk and nanostructured form.**Temperature-dependent specific heat capacity of cobalt monoxide in bulk form and as thin films with 10 nm grain size. The data were implemented in COMSOL Multiphysics ® to model the thermal response of nanocalorimetric measurements. Figure adapted from Tang et al.^2^

**S3.- Heat capacity analysis.**

The heat capacity analysis in the nanocalorimetric technique based on microsecond pulse heating builds upon the quasi-adiabatic approach, leveraging the possibility of linearizing the temporal evolution of the relevant variables. For completeness, we include in the supplementary material a detailed analysis of the quasi-adiabatic method, which has been extensively described in the literature^1,3^.

In membrane-based nanocalorimetric devices, the calorimetric cell incorporates a thin metallic layer that functions simultaneously as a resistive heater and a temperature sensor. This configuration enables the delivery of thermal power via Joule heating and allows for the measurement of the average temperature of the cell during thermal scans.

Throughout the heat capacity analysis, it will be assumed that temperature gradients within the calorimetric cell are moderate, thereby justifying the use of the average temperature as a representative value for the system, nevertheless, this assumption has limitations. Due to the reduced out-of-plane dimensions of both the calorimetric cell and the sample, the in-plane thermal conductance is inherently limited. As previously evaluated^4,5^, the spatial temperature distribution is highly dependent on the applied heating rate and the presence of additional thermal diffusion layers within the calorimetric structure. When the heating rate is low compared to the characteristic lateral cooling rates, significant inhomogeneities can arise in the two-dimensional temperature distribution, typically resulting in parabolic temperature profiles. Conversely, under high heating rates, the temperature distribution becomes significantly flatter, thereby validating the average temperature approximation. This assumption is further supported by finite element modeling and experimental observations^27^, which show that for typical heater geometries, whether meander-like or strip-like, and sensing areas on the millimeter scale, temperature inhomogeneities remain within approximately 20 K at heating rates on the order of 10^5^ K/s.

In the quasi-adiabatic method, nanocalorimeters are driven by short pulses of constant current, sufficiently intense to induce heating rates that far exceed the characteristic cooling rate of the system. By locally measuring the voltage drop across the calorimetric cell using a four-wire configuration, one can determine the instantaneous power input as $P_{in}\left( t \right)=V\left( t \right)\cdot I\left( t \right)$, as well as the time-dependent resistance $R\left( t \right)={V\left( t \right)}/{I\left( t \right)}$. Using a prior calibration of the resistance-temperature relationship, the temperature evolution $T(R(t))$, can be retrieved, and consequently, the effective heating rate can be calculated as, $\beta\left( t \right)={dT\left( t \right)}/{dt}$.

A straightforward analysis leads to the conclusion that the apparent heat capacity of a single calorimetric cell, ${Cp}_{\beta}^{cc}\left( t \right),$can be expressed as:

$${Cp}_{\beta}^{cc}\left( t \right)=\frac{P_{in}\left( t \right)}{\beta\left( t \right)}={Cp}_{real}^{cc}\left( t \right)+\frac{P_{losses}\left( t \right)}{\beta\left( t \right)}$$

where $P_{losses}$ term includes contributions from thermal conduction through the membrane and radiative heat losses, and thus, accounts for the energy lost per unit time due to non-adiabatic effects. Power losses depend primarily on the temperature difference between the calorimetric cell and its surroundings, and also on the internal temperature distribution within the cell at a given time. To ensure quasi-adiabatic conditions, the input power $P_{in}$ must be significantly greater than $P_{losses}$, thereby minimizing the influence of thermal losses. As noted by Efremov et al. ^1^, a first-order estimation of $P_{losses}$can be performed for each calorimetric cell by measuring the apparent heat capacity at different heating rates. The plot of ${Cp}_{\beta}^{cc}\left( T \right)$ as a function 1/*β* at a fixed temperature allows for the extrapolation of the real heat capacity ${Cp}_{real}^{cc}$ as the intercept, and the loss term $P_{losses}$ as the slope. Nevertheless, the approximation of adiabatic behaviour becomes valid for most practical scenarios - such as at high heating rates or within a narrow temperature scan range - the apparent heat capacity ${Cp}_{\beta}^{cc}$ closely matches the real heat capacity ${Cp}_{real}^{cc}$. In this framework, the measurement of a sample requires two consecutive measurements of the same calorimetric cell: first with the empty CC (denoted with superscript 0) and subsequently with the sample evaporated onto it (denoted with superscript 1).

$${C_{P}}^{sample} \left( T \right)\approx{C_{P}}_{\beta}^{cc,1}\left( T \right)-{C_{P}}_{\beta}^{cc,0}\left( T \right)$$

Analysing the intrinsic noise of this measurement reveals that it is largely dominated by the inefficiency of computing the time derivative of a digitized signal. The apparent heat capacity is obtained by measuring the voltage drop across the calorimetric cell, $V_{S}$, and across a load resistor, $V_{I}$, which is used to determine the current supplied to the device $I(t)$. These signals are acquired using a 16-bit data acquisition system (DAQ) operating at a given sampling frequency $f$. In the best-case scenario when utilizing the full dynamic range of the DAQ, the base digital noise used to be equivalent to approximately three times the least significant bit (LSB), and then the signal-to-noise ratio (SNR) (defined as $SNR=V/{\eta_{V}}$, where $\eta_{V}$ is the root mean square (RMS) noise of the signal $V$) remains below 2x10^4^.

The uncertainty in the input power term, $\eta_{P}$, in the expression for apparent heat capacity can be estimated as:

$\eta_{P}={\sqrt{2}\cdot V\cdot\eta_{V}/R}_{\text{load}}$,

assuming that $R_{load}$ is typically chosen such that $V_{I}\sim V_{S}$, and therefore both signals share a similar noise level $\eta_{V}$.

Similarly, considering the acquisition frequency $f$ and the temperature coefficient of resistance $\alpha\cdot R_{o}$, the propagated uncertainty in the heating rate measurement can be expressed as:

$$\eta_{B}=\sqrt{2}\cdot\frac{\eta_{v}\cdot R_{load}}{{V\cdot R}_{o}\cdot\alpha}\cdot f\approx\frac{\sqrt{2}\cdot f}{SNR\cdot\alpha}$$

By rewriting the expression, it becomes evident that a higher signal-to-noise ratio (SNR) in the voltage measurement leads to a lower uncertainty $\eta_{B}$. However, the presence of the acquisition frequency $f$ in the numerator of the expression highlights the significant impact that differentiating digitized signals has on the uncertainty, thereby fundamentally limiting the precision of the method. A traditional strategy used in instrumentation to overcome this lack of precision is to consider differential measurements. Using a couple of twin calorimetric cells working simultaneously, differential signatures can be widely amplified while fitting the range of the ADC card. By this approach we can effectively reach the limit of the intrinsic thermal noise (or Johnson-Nyquist noise) of the metallic elements used as thermometer and with the pre-amplification stage.

The differential methodology can be implemented by connecting the nanocalorimeters in series and performing the differentiation using instrumentation amplifiers (as shown in Figure 2 (a) of the article).

The differential voltage drop between the two calorimetric cells, defined as $\Delta V=V_{S}-V_{R}$, where cell S hosts the sample and cell R serves as the reference, can be acquired with high amplification (typically ×10 to ×1000) using an instrumentation amplifier. The difference in apparent heat capacity between the two cells can then be recalculated by incorporating the differential voltage ∆V into the general expression:

$${\Delta C}_{P}\left( T_{S}(t) \right)=\frac{P_{S}}{\beta_{S}}-\frac{P_{R}}{\beta_{R}}$$

Note that the differential heat capacity between both devices, ${\Delta C}_{P}\left( T_{S}(t) \right)$, is a sample temperature function, which in turn is a time function. For convenience, this dependence is not shown explicitly.

As a first step we can express the power terms as function of the voltage dropped in the calorimetric cells:

$${\Delta C}_{P}=\frac{V_{S} \cdot I}{\beta_{S}}-\frac{V_{R}\cdot I}{\beta_{R}}=\frac{I {\cdot V}_{R}}{\beta_{R}} \left( \frac{V_{S}\cdot\beta_{R}}{V_{R}\cdot\beta_{S}}-1 \right)$$

We use the differential configuration relation, $V_{S}=V_{R}+\Delta V$, to obtain

$${\boldsymbol{\Delta}C}_{P}=\frac{I\cdot V_{R}}{\beta_{R}} \left[ \frac{\left( {\Delta V+V}_{R} \right)\cdot\beta_{R}}{V_{R}\cdot\beta_{S}}-1 \right]=\frac{I\cdot\Delta V}{\beta_{S}}+\frac{{I\cdot V}_{R}}{\beta_{S}} \left( 1-\frac{\beta_{S}}{\beta_{R}} \right)$$

As in the development of Efremov et al.^1^ we spread the expression for .${d\Delta V}/{dt}$:

$$\Delta V=V_{S}-V_{R}\to\frac{d\Delta V}{dt}=\frac{{dV}_{S}}{dt}-\frac{{dV}_{R}}{dt}$$

If functions $V(t)$ and $T(t)$ are derivative and continuous, then ${dV}/{dt}=({dV}/{dT})({dT}/{dt})$. Using this relation on previous equation) and by reordering we can write:

$$\frac{d\Delta V}{dt}=\frac{dV_{S}}{dT_{S}} \beta_{S}-\frac{dV_{R}}{dT_{R}} \beta_{R} \to\frac{\beta_{S}}{\beta_{R}}=\frac{\left( {d\Delta V}/{dt} \right)_{t}}{{\beta_{R} \left( {dV_{S}}/{dT_{S}} \right)}_{t}}+\frac{\left( {dV_{R}}/{dT_{R}} \right)_{t}}{\left( {dV_{S}}/{dT_{S}} \right)_{t}}$$

By substituting the equivalent expression for the ratio between heating rate, we obtain:

$${\boldsymbol{\Delta}C}_{P}\left( T_{S}(t) \right)=\frac{I\cdot\Delta V}{\beta_{S}}+\frac{{I\cdot V}_{R}}{\beta_{S}} \left[ 1-\left( \frac{\left( {d\Delta V}/{dt} \right)_{t}}{\beta_{R} \left( {dV_{S}}/{dT_{S}} \right)_{t}}+\frac{\left( {dV_{R}}/{dT_{R}} \right)_{t}}{\left( {dV_{S}}/{dT_{S}} \right)_{t}} \right) \right]$$

And finally considering that current injected is constant in time, then $V_{i}=I_{i} R_{i} -\to d\left( V_{i} \right)=d\left( I_{i} R_{i} \right)=I_{i} d\left( R_{i} \right)$ and can be written in the expression that is routinely used in quasi-adiabatic experiments and has been adopted in microsecond pulsed experiments:

$${\boldsymbol{\Delta}C}_{P}\left( T_{S}(t) \right)=\frac{I \Delta V}{\beta_{S}}-\frac{V_{R}}{\beta_{S} \beta_{R}} \frac{\left( {d\Delta V}/{dt} \right)_{t}}{\left( {dR_{S}}/{dT_{S}} \right)_{t}}+\frac{V_{R} I}{\beta_{S}}\left[ 1-\frac{\left( {dR_{R}}/{dT_{R}} \right)_{t}}{\left( {dR_{S}}/{dT_{S}} \right)_{t}} \right]$$

By selecting nanocalorimeters from the same wafer, it is possible to obtain twin pairs of devices in which differences in initial resistance and temperature coefficient of resistance (TCR) are practically negligible. Under these conditions, the first and last terms of the equation will be practically zero during the early stages of the pulse. If samples with heat capacities comparable to the addenda are incorporated, the cell containing the sample will heat at a slightly lower rate than the empty one, and the first term, which includes ΔV, will increase for longer pulse durations. Among all the terms, the second term contains most of the information about the difference in heat capacity and is intrinsically somewhat noisier since it includes the time derivative of the voltage difference. In Figure S5 we present a differential measurement performed on devices with a meander-shaped heater/sensor with a sensing area of 1 mm², where a thin indium layer of 40 nm has been deposited. Starting the pulse at 320 K, we can observe the evolution of the three terms, clearly showing, as explained above, that during the initial stages the central term dominates.

**Figure S6.** Heat capacity of 40 nm of indium, decomposed in the three terms obtained.

**S4.- Experimental validation of transient response**

The process of scaling down is not entirely free from challenges. In the initial implementations, we used devices with long strip heaters, several millimetres in length, which exhibited inductive effects that hindered accurate temperature sensing during the early stages of the pulse. Geometrical analysis of the heater’s self-inductance showed that it is proportional to the strip length and to the logarithm of the inverse of its transverse dimensions, namely width and thickness. This means that longer strips, or those narrower or thinner, exhibit stronger inductive behaviour.

**Figure S7.** Minimizing Inductive Artifacts in Miniaturized Nanocalorimeter Designs. Voltage response during a single pulse for devices of different sizes and configurations, highlighting the improved signal linearity in the optimized design. (Red curve): Original 50 × 50 µm² strip-type heater layout showing inductive distortion due to extended lead length. (Black curve): Optimized design with shortened leads, effectively suppressing inductive effects and enhancing signal linearity.

Since the strip width defines the calorimetric cell size and is fixed by design, we addressed this issue by increasing the metal thickness and, more effectively, by reducing the strip length, guided by the thermal diffusion constraints. Figure S7 shows the voltage response measured in a four-point configuration for two 50 × 50 µm strip-type calorimeters: one with a long strip several millimeters in length, and another with a short strip of 150 µm. The long-strip design exhibits an inductive transient lasting 150 µs, whereas in the short-strip design this transient is reduced to less than 10 µs, enabling accurate temperature sensing from the onset of the pulse.

As mentioned in the main text, the calorimetric signal is initially dominated by the inductance of the electronic chain combined with that of the devices and contacts. In a second stage, capacitive components become dominant. Using twisted wires inside the cryostat and BNC connectors for external connections ensures capacitances below 100 pF/m. Considering the complete setup characteristics, with wiring lengths below 5 m and a total circuit resistance under 100 Ω, the resulting RC time constants are on the order of tens of nanoseconds. Nevertheless, the capacitive component observed in the experimental curves reveals transient responses lasting a few microseconds, which likely arise from the combined effect of the intrinsic nanocalorimeter capacitance and the complete electronic chain, including the Howland current source and the amplifier. This prevents reducing the pulse duration below that range.

As discussed in the main text, selecting an averaging measurement window after the transient ensures accurate evaluation of the calorimetric signal.

**S5.- Device geometry and dimensions.**

This section provides detailed geometrical dimensions of the nanocalorimetric devices used in this study to facilitate reproducibility and comparison with future designs.

**Figure S8. Geometrical layouts and optical images of the fabricated nanocalorimetric devices.** Top panels (a,b): schematic top views of the suspended nanocalorimeters used in this work, showing the membrane geometry, supporting beams, electrical leads, and central heater–thermometer region for the different device designs. Bottom panels (c-e): optical microscope images of the corresponding fabricated devices, including the meander-type calorimeter and the miniaturized strip-type calorimeters. Scale bars are indicated in each image.

The designs illustrate the different device layouts employed to explore the effect of lateral size and geometry on thermal diffusion and calorimetric performance. In the schematic layouts, blue regions correspond to the silicon frame, white regions to the suspended silicon nitride membrane, and gray regions to the patterned Ti/Pt metallic elements, which act simultaneously as heater, thermometer, and electrical leads. The bottom optical images provide a direct view of the real fabricated devices, including the meander-type geometry and the reduced-area strip-type geometries used for the µs-pulsed heating measurements. These images complement the schematic layouts and allow a clearer visualization of the different device architectures discussed in the main text.

**References**

1.	Efremov, M. Y. *et al.* Thin-film differential scanning nanocalorimetry: heat capacity analysis. *Thermochim. Acta* **412**, 13–23 (2004).

2.	Tang, Y. J. *et al.* Finite size effects on the moment and ordering temperature in antiferromagnetic CoO layers. *Phys. Rev. B* **67**, 054408 (2003).

3.	Rodríguez-Viejo, J. & Lopeandía, A. F. Quasi-adiabatic, Membrane-Based, Highly Sensitive Fast Scanning Nanocalorimetry. *Fast Scanning Calorim.* 105–149 (2016) doi:10.1007/978-3-319-31329-0_3.

4.	Lopeandia, A. F. *et al.* Heat transfer in symmetric U-shaped microreactors for thin film calorimetry. *J. Micromechanics Microengineering* **16**, 965–971 (2006).

5.	Lopeandia, A. F. Development of Membrane-based Calorimeters to Measure Phase Transitions at the Nanoscale. *PhD thesis. Univ. Autònoma Barcelona.* (2007).
